# Modulation in serum and hematological parameters as a prognostic indicator of COVID-19 infection in hypertension, diabetes mellitus, and different cardiovascular diseases

**DOI:** 10.3389/fchem.2024.1361082

**Published:** 2024-04-29

**Authors:** Muhammad Ishtiaq Jan, Riaz Anwar Khan, Naeem Khan, Syed Muhammad Iftikhar, Sajid Ali, M. I. Khan, Saima Gul, Umar Nishan, Tahir Ali, Riaz Ullah, Ahmed Bari

**Affiliations:** ^1^ Department of Chemistry, Kohat University of Science and Technology, Kohat, Khyber Pakhtunkhwa, Pakistan; ^2^ Qazi Hussain Ahmad Teaching Hospital, Nowshehra, Khyber Pakhtunkhwa, Pakistan; ^3^ Department of Chemistry, Bacha Khan University, Charsadda, Khyber Pakhtunkhwa, Pakistan; ^4^ State Key Laboratory of Chemical Oncogenomics, Guangdong Provincial Key Laboratory of Chemical Genomics, Peking University Shenzhen Graduate School, Shenzhen, Guangdong, China; ^5^ Department of Pharmacognosy, College of Pharmacy, King Saud University, Riyadh, Saudi Arabia; ^6^ Department of Pharmaceutical Chemistry, College of Pharmacy, King Saud University, Riyadh, Saudi Arabia

**Keywords:** COVID-19, D-dimer, ferritin, hs-CRP, and LDH

## Abstract

SARS-CoV-2 infection affects and modulates serum as well as hematological parameters. However, whether it modifies these parameters in the existing disease conditions, which help in the erection of specific treatments for the disease, is under investigation. Here, we aimed to determine whether serum and hematological parameters alteration in various diseases, diabetes mellitus (DM), hypertension (HTN), ischemic heart disease (IHD) and myocardial infarction (MI) conditions correlate and signal SARS-CoV-2 infection, which could be used as a rapid diagnosis tool for SARS-CoV-2 infection in disease conditions. To assess the projected goals, we collected blood samples of 1,113 male and female patients with solo and multiple disease conditions of DM/HTN/IHD/MI with severe COVID-19, followed by biochemical analysis, including COVID-19 virus detection by RT-qPCR. Furthermore, blood was collected from age-matched disease and healthy individuals 502 and 660 and considered as negative control. In our results, we examined higher levels of serum parameters, including D-dimer, ferritin, hs-CRP, and LDH, as well as hematological parameters, including TLC in sole and multiple diseases (DM/HTN/IHD/MI) conditions compared to the control subjects. Besides, the hematological parameters, including Hb, RBC, and platelet levels, decreased in the patients. In addition, we found declined levels of leukocyte count (%), lymphocyte (%), monocyte (%), and eosinophil (%), and elevated level of neutrophil levels (%) in all the disease patients infected with SARS-CoV-2. Besides, NLR and NMR ratios were also statistically significantly (*p* < 0.05) high in the patients with solo and multiple disease conditions of DM/HTN/IHD/MI infected with the SARS-CoV-2 virus. In conclusion, rapid alteration of sera and hematological parameters are associated with SARS-CoV-2 infections, which could help signal COVID-19 in respective disease patients. Moreover, our results may help to improve the clinical management for the rapid diagnosis of COVID-19 concurrent with respective diseases.

## Introduction

The Severe Acute Respiratory Syndrome Coronavirus-2 (SARS-CoV-2) pandemic has devastated humanity since its outbreak in 2019 (Chekol Abebe et al., 2022). Great efforts have been made to describe its pathophysiology and develop more effective treatments (Wang and Yang, 2023; Yang and Wang, 2023) In addition to the lungs, the virus has been proven to damage several other systems, including the heart, gastrointestinal tract, neurological system, kidney, coagulation, and endothelium (Iba et al., 2020; Puelles et al., 2020). However, a number of hematological parameters, such as lymphopenia and increased D-dimers, have been observed in COVID-19 patients (Rahi et al., 2021). Several studies have consistently indicated that higher red blood cell distribution width predicts worse outcomes in individuals with COVID-19 infection. However, anemia has been reported to be an independent predictor of disease severity in COVID-19 infection (Bellmann-Weiler et al., 2020; Higgins et al., 2020; Banon et al., 2021).

Red blood cells (RBC) are the carriers of oxygen throughout the body and are known to lyse in cases of infection (Bouchla et al., 2022), especially with the increasing complications of sepsis (Bateman et al., 2017). Previous studies have shown that the virus can invade erythrocytes by interacting its S1 spike protein with erythrocyte CD147 (Al-Salih et al., 2020) and the band-3 protein (Cosic et al., 2020). However, viral spike proteins and complement activation products have been detected on the cell surface of erythrocytes from patients with COVID-19. Therefore, it is thought to affect erythrocyte rheology, leading to intravascular thrombosis and associated lung injury (Lam et al., 2021).

Additionally, the hypercoagulation and inflammatory state lead to more fragility and make elastic the membrane of RBC (Grobler et al., 2020). It has been demonstrated that high serum ferritin levels in COVID-19 patients might result in hyper-activated interactions between erythrocytes and platelets (Youssry et al., 2022), indicating a connection between erythrocyte alterations and thrombosis in COVID-19 disease (Venter et al., 2020).

The understanding about the immune response of SARS-CoV-2 has been studied in detail in different cohort studies. Moreover, due to the significant sequence homology throughout the coronavirus family, several scientists have hypothesized the interaction between SARS-CoV-2 and the host organism may be related to another coronavirus (Vabret et al., 2020). Previously, the immunological dysfunction caused by SARS-CoV-2 confirmed the activation of innate and adaptive immune responses (Yang et al., 2004; Chu et al., 2016). Therefore, viral infection may have damaged the immune system and may be reflected as a change in peripheral blood cells.

Lymphocytes are essential for maintaining immunological homeostasis during infection (Channappanavar et al., 2014). Several studies have shown that lymphopenia can predict longevity in COVID-19 patients (Yang et al., 2004; Channappanavar et al., 2014) Additionally, numerous investigations have revealed that eosinophilia is associated with poor prognosis. (Cortés-Vieyra et al., 2021; Singh et al., 2022). Thus, immunological damage at an early stage of the disease may be indicated by the differentiation of peripheral leukocytes. However, modulation in the levels of eosinophils are the risk factors for changes in peripheral blood cells (Tong et al., 2021).

This study aimed to analyze the effect of COVID-19 on serum and hematological parameters in, DM, HTN, and different cardiovascular diseases before a detailed investigation as a rapid diagnostic tool to reduce the risk of mortality in respective patients.

## Material and methods

Data was collected from 1,113 hospitalized patients, including 495 males and 618 females, in the intensive care unit of Qazi Hussain Ahmad Teaching Hospital, Nowshehra, Pakistan, due to severe COVID-19 disease with SpO_2_ levels less than 75 mmHg, from April 2020 to January 2023. In addition, earliest clinical manifestations such as fever, cough, dyspnea, muscle pain, diarrhea fatigue, and loss of smell and taste recorded in medical history taken upon admission. Furthermore, in the present research work only those patients and normal individuals were considered who were not previously vaccinated. Herein, two control groups were used to better understand the results and their blood was collected from respective departments. Control group 1 was composed of 502 male and female patients with DM, HTN, IHD and MI without COVID-19. However, in control group 2, 660 normal individuals, including 305 males and 355 females, were included without DM, HTN, IHD, MI and COVID-19. Furthermore, patients with renal diseases, hepatic dysfunction, anemia, myeloid tumor, or other diseases were excluded. Whole blood and serum samples were obtained from all participants in EDTA and serum tubes under the supervision of expert and qualified medical physician and standard operating procedures in the hospital. Laboratory investigations, chest CT imaging, and SpO_2_ levels were performed immediately after the hospitalization of patients. The present study was approved by the ethical committee of Kohat University of Science and Technology, Kohat, Pakistan under Reference No: Ref-No/KUST/Ethical Committee/528. In this study, written informed consent was obtained from all participants. The study was conducted following the guidelines of the Declaration of Helsinki (World Medical Association declaration of Helsinki, 2013: Ethical principles for medical research involving human subjects, 2013).

### Hematological and biochemical analysis

An appropriate history and detailed clinical examination were recorded for each patient. Blood samples were collected at the time of admission to the hospital. However, various hematological investigations, including total leukocyte count (TLC), neutrophils, lymphocytes, monocytes, eosinophils, basophils, RBC, hemoglobin (Hb), and platelet count were investigated using Sysmex XP-300 automated analyzer. The standard biochemical analysis of serum components glucose, glycohemoglobin (HbA1c), urea, creatinine, serum glutamyl pyruvate transaminase (SGPT), alkaline phosphatase (ALP) and total bilirubin was performed according to the protocol performed in the routine examination in the hospital. D-dimer (D-Dimer Human ELISA Kit, Cat # EHDDIMER), ferritin (Ferritin Human ELISA Kit, Cat # EHFTL), high-sensitive C reactive protein (hs-CRP) (Boster Bio ELISA KIT, Cat # EK7040), Lactate dehydrogenase (LDH) (L- LDH reagent kit, Cat # TR20015), were performed both in the patients and the control groups according to the instructions provided with the kit.

### RT-qPCR analysis

All the patients and control groups were investigated for C0VID-19 infection through RT-qPCR. In addition, only those patients who showed positive results were considered for further investigation while in the control groups who showed negative results were selected for further study. The RNA was collected through the MagMAX™ Viral/Pathogen Nucleic Acid Isolation Kit, Cat # A42352, Applied Biosystems, using the instructions provided with the kit. cDNA was synthesized from the RNA of the COVID-19 virus with the High-Capacity RNA-to-cDNA™ Kit, Cat #: 4387406, Applied Biosystems, using the instructions provided with the kit. cDNA was amplified through RT-qPCR with SYBR™ Select Master Mix, Cat #: 4472913, Applied Biosystems, using the instructions provided with the kit.

### Statistical analysis

The SPSS 21 service (IBM Corp., Armonk, NY, United States) was used to analyze statistics among different groups using ANOVA followed by Tukey’s multiple comparison test. In addition, Student’s t-test was performed for comparison between the two groups. However, qualitative analysis was performed using Mann-Whitney t-test. All the data is presented as the mean ± SD. *p < 0.05 was considered to be statistically significant.

## Results

**FIGURE 1:**
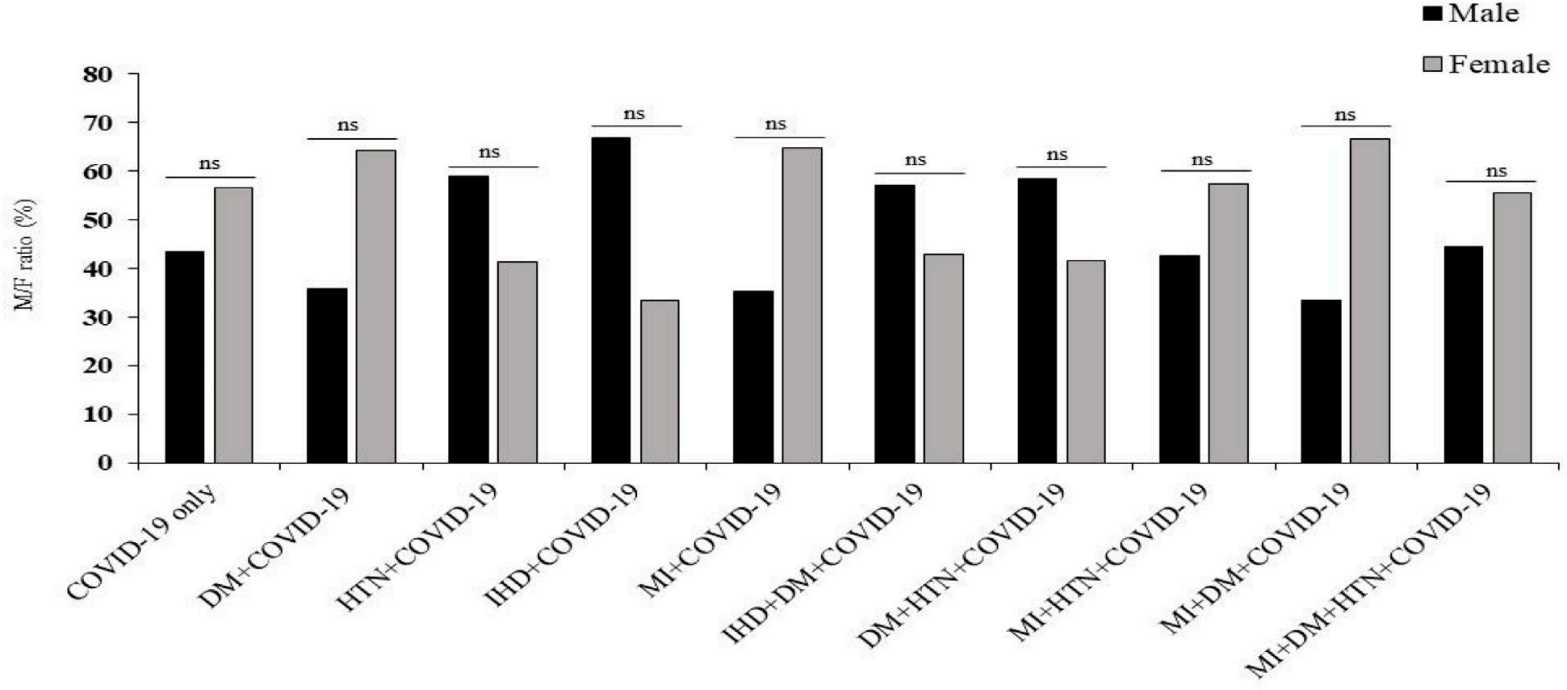
Prevalence of COVID-19 infection in male and female patients with DM, HTN, IHD and MI. δ Mann-Whitney t-test. *p-value was significant when less than 0.05. ns: non-significant.

In this study, hospitalized SARS-CoV-2 patients with different diseases, including COVID-19 only, DM, HTN, and other cardiovascular diseases, were included. In addition, an age and gender-matched healthy control group for each disease group was also included. The demographic characteristics showed that the ratio of COVID-19 was high in female patients of COVID-19 only, DM, MI, MI with HTN, MI with DM, and MI with DM and HTN patients. In addition, the ratio of COVID-19 was high in male patients in the HTN, IHD, IHD with DM, and DM with HTN groups given in Figure 1.

### Serum markers

**FIGURE 2:**
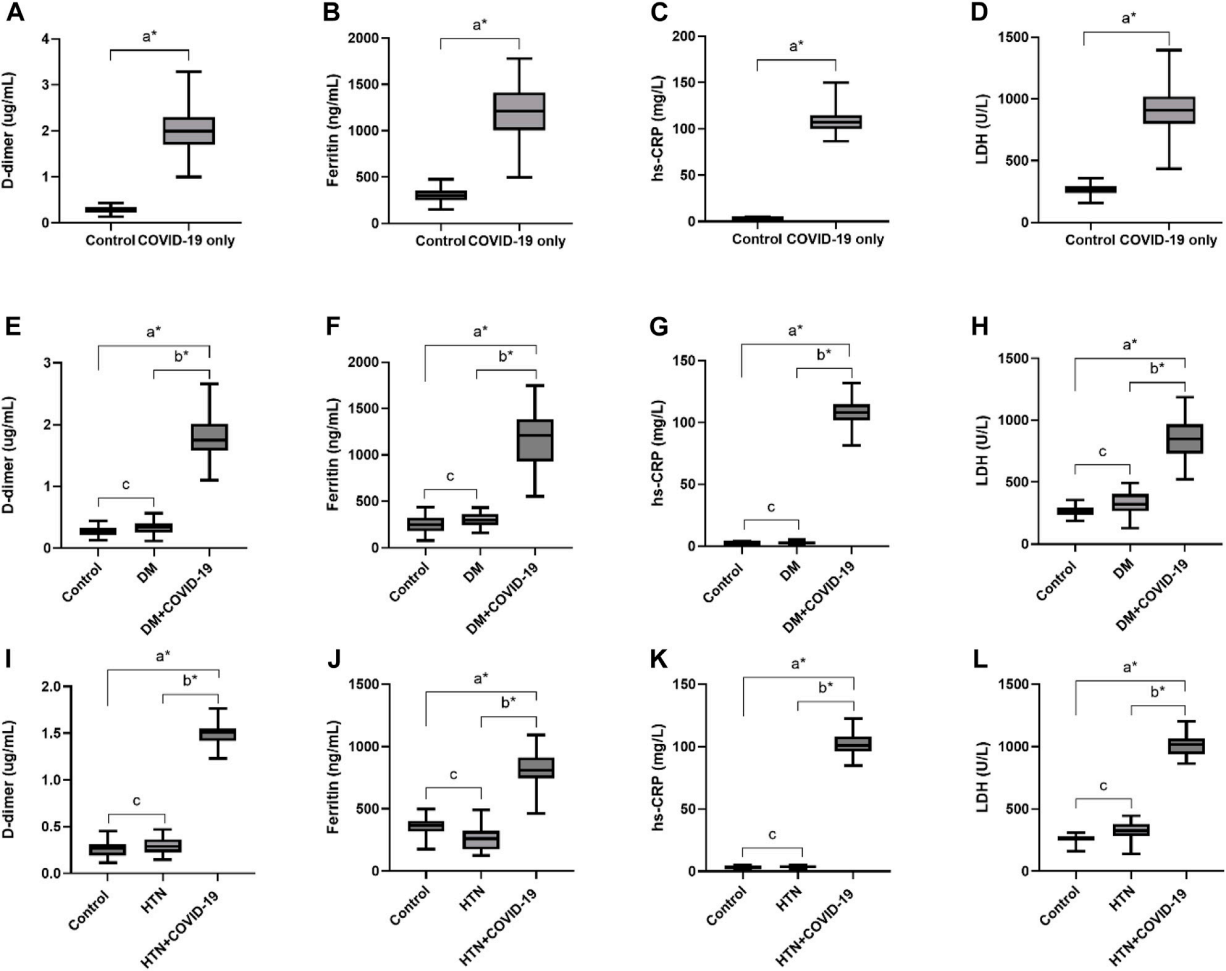
Increased levels of D-dimer, Ferritin, hs-CRP and LDH in COVID-19 only and COVID-19 patients with DM and HTN. **(A)**, D-dimer. **(B)**, Ferritin, **(C)**, hs-CRP and **(D)**, LDH levels in COVID-19 patients only. **(E)**, D-dimer. **(F)**, Ferritin, **(G)**, hs-CRP and **(H)**, LDH levels in diabetes mellitus patients infected with COVID-19. **(I)**, D-dimer. **(J)**, Ferritin, **(K)**, hs-CRP and **(L)**, LDH levels in hypertensive patients suffered from COVID-19 infection. δ *p value was significant when less than 0.05 ^a^ Disease with COVID-19 vs healthy control ^b^ Disease with COVID-19 vs disease control ^c^ Disease without COVID-19 vs healthy control.

In this cohort study, we have thoroughly investigated and compared patient characteristics among the patients with severe COVID-19 disease. D-dimer is one of the investigations performed in COVID-19 patients to detect thrombosis. However, previous studies showed that a 3 to 4-fold increase in D-dimer levels is associated with a better prognosis in COVID-19 patients (Yao et al., 2020). In the present finding, the levels of D-dimer were significantly increased in patients with COVID-19 only, and solo disease condition of DM, and HTN infected with COVID-19 (2 ± 0.55, 1.80 ± 0.34 and 1.50 ± 0.12 μg/mL, respectively) compared to control. Ferritin is recognized as a critical mediator of immune system irregularities, particularly in cases of severe hyperferritinemia, through direct immune suppression and pro-inflammatory activities. It helps create cytokine storms that damage many organs. However, serum ferritin is also a unique severity risk factor in COVID-19 patients (Vargas-Vargas and Cortés-Rojo, 2020). In the present finding, the serum ferritin level was significantly high in the respective COVID-19 patients (1,191.39 ± 332.15, 1,142.21 ± 272.73, 812.82 ± 142.81 μg/L respectively). The significantly elevated level of hs-CRP was also observed in COVID-19 patients with the values 107.29 ± 10.26, 109.5 ± 9.27 and 102.96 ± 7.96 mg/L, respectively. Different clinical disorders, including hemolysis, cancer, severe infections, sepsis, and liver illnesses, have high serum LDH levels. The progression of COVID-19 may also be associated with LDH elevation. The severity and status of the disease may therefore be correlated with LDH levels (Wu et al., 2020). Herein, it has also been shown that the LDH levels were significantly higher in patients who were infected with COVID-19 only and solo disease condition of DM and HTN infected with COVID-19 (896.2 ± 167.23, 860.07 ± 167.63 and 1,009.65 ± 99.68 U/L) when compared with controls (Table 1 and Figures 2A–L).

**TABLE 1:** Clinical characteristics of patients infected with COVID-19 only and patients of DM and HTN infected with COVID-19.

	COVID-19 only	Control	*p-*value	DM + COVID-19	DM	Control	*p-*value	HTN + COVID-19	HTN	Control	*p-*value
M/F	203/264	88/126		80/144	44/28	51/73		40/28	40/23	31/19	
M/F %	43.4/56.6	41.1/58.9		35.7/64.3	61/39	41.1/58.9		58.8/41.2	63/37	62/38	
Age (Year)	59.5 ± 9.6 (35,90)	60 ± 12.87 (26,82)	^e,^0.94	64.15 ± 10.51 (30,89)	60.66 ± 11.88 (33,80)	65.12 ± 12.62 (26,79)	^a^ ^,a^0.73 ^a^ ^,b^0.09	62.30 ± 15.12 (29,79)	58.19 ± 16.29 (27,83)	62.02 ± 14.74 (33,80)	^a^ ^,a^0.98,^a^ ^,b^0.09
Systolic (mmHg)	119.63 ± 9.24 (110,150)	124.41 ± 10.9 (105,145)	^e,^<0.00^b^	135.18 ± 9.23 (110,145)	122.37 ± 11.02 (103,155)	119.6 ± 9.12 (105,135)	^a^ ^,^ ^c^<0.0^b^,^b^0.07	190.12 ± 14.65 (160,210)	192.42 ± 14.48 (155,215)	120.60 ± 7.93 (105,135)	^a^ ^,^ ^c^<0.00^b^,^a^ ^,^ ^d^ <0.00^b^
Diastolic (mmHg)	86.19 ± 6.13 (75,95)	80.14 ± 6.27 (70,90)	^e,^<0.00^b^	85.09 ± 4.18 (80,95)	78.59 ± 6.21 (70,95)	80.12 ± 6.33 (70,90)	^a^ ^,^ ^c^<0.00^b^,^a^ ^,^ ^d^<0.00^b^	101.47 ± 7.48 (90,110)	102.63 ± 7.15 (90,120)	79.50 ± 6.64 (70,90)	^a^ ^,^ ^c^<0.00,^a^ ^,^ ^d^<0.00^b^
D Dimer (µg/mL)	2 ± 0.55 (1.1,3)	0.27 ± 0.08 (0.14,0.42)	^e,^<0.00^b^	1.80 ± 0.34 (1.2,2.3)	0.33 ± 0.11 (0.14,0.57)	0.27 ± 0.08 (0.13,0.4)	^a^ ^,^ ^c^<0.00^b^ ^a^ ^,b^0.39	1.50 ± 0.12 (1.4,1.7)	0.30 ± 0.08 (0.16,0.49)	0.26 ± 0.08 (0.13,0.4)	^a^ ^,^<0.00^b^ ^a^ ^,b^0.41
Ferritin (ng/mL)	1,191.39 ± 332.15 (553.1, 1710)	311.9 ± 84.55 (187,432)	^e,^<0.00^b^	1,142.21 ± 272.73 (781,1631)	298.63 ± 89.62 (78,430)	309.15 ± 84.05 (80,435)	^a^ ^,^ ^c^<0.00^b^,^a^ ^,b^0.97	812.82 ± 142.81 (681,1090)	299.65 ± 97.79 (125,490)	357.08 ± 74.62 (177,422)	^a^ ^,^ ^c^<0.00^b^,^a^ ^,b^0.86
RBS (mg/dL)	146.68 ± 25.6 (102,189)	161.68 ± 26.59 (120,198)	^e,^<0.00^b^	428.64 ± 66.21 (319,561)	162.23 ± 25.36 (120,198)	160.78 ± 26.61 (114,195)	^a^ ^,^ ^c^<0.00^b^,^a^ ^,b^0.95	171.06 ± 10.5 (143,176)	167.34 ± 23.71 (120,198)	163.64 ± 24.77 (110,192)	^a^ ^,a^1.00,^a^ ^,b^1.00
FBS (mg/dL)	85.99 ± 7.69 (71,101)	89.74 ± 6.78 (76,98)	^e,^<0.00^b^	199.71 ± 6.80 (189,209)	87.72 ± 9.75 (67,107)	89.44 ± 6.72 (80,103)	^a^ ^,^ ^c^<0.00^b^,^a^ ^,b^1.00	92.00 ± 5.89 (80,98)	92.39 ± 8.79 (78,109)	89.46 ± 7.5 (74,101)	^a^ ^,a^0.99,^a^ ^,b^0.98
HbA1c (%)	5.02 ± 0.51 (4,6)	4.86 ± 0.58 (3.8,5.6)	^e,^0.99	10.24 ± 2.07 (7,14.1)	4.82 ± 0.62 (3.80,5.60)	4.85 ± 0.58 (3.5,5.4)	^a^ ^,^ ^c^<0.00^b^,^a^ ^,b^1.00	5.53 ± 0.7 (5,7)	4.51 ± 0.73 (3.3,5.6)	4.69 ± 0.71 (3.4,5.9)	^a^ ^,a^0.81,^a^ ^,b^0.99
Blood Urea (mg/dL)	67.4 ± 41.8 (21,368)	38.63 ± 20.78 (26,68)	^e,^<0.00^b^	68.29 ± 42.94 (23,180)	39.8 ± 20.78 (21,70)	38.91 ± 20.94 (22,63)	^a^ ^,^ ^c^<0.00^b^,^a^ ^,b^0.78	39.88 ± 16.04 (23,61)	43.29 ± 20.81 (19,72)	41.26 ± 21.41 (21,70)	^a^ ^,a^0.76,^a^ ^,b^0.87
CRP mg/L	107.29 ± 10.26 (90,121)	2.73 ± 0.63 (1.9,4)	^e,^<0.00^b^	109.5 ± 9.27 (92,124)	2.806,111 ± 1.47 (0.20,5.30)	2.73 ± 0.63 (1.8,3.9)	^a^ ^,^ ^c^<0.00^b^,^a^ ^,b^0.97	102.96 ± 7.96 (101,123)	3.28 ± 0.99 (1.4,4.9)	2.94 ± 0.75 (2,4.8)	^a^ ^,^ ^c^<0.00^b^,^a^ ^,b^0.54
S LDH U/L	896.2 ± 167.23 (434,1201)	266.7 ± 40.51 (194,309)	^e,^<0.00^b^	860.07 ± 167.63 (561,1123)	319.68 ± 97.92 (129,493)	267.69 ± 40.1 (189,319)	^a^ ^,^ ^c^<0.00^b^,^a^ ^,b^0.14	1,009.65 ± 99.68 (891,1120)	314.52 ± 77.65 (138,409)	262.86 ± 5.01 (195,310)	^a^ ^,^ ^c^<0.00^b^,^a^ ^,b^0.45
Creatinine (mg/dL)	1.77 ± 0.83 (0.39,3.7)	0.88 ± 0.41 (0.2,1.7)	^e,^<0.00^b^	1.32 ± 0.85 (0.3,3.3)	0.85 ± 0.41 (0.20,1.70)	0.90 ± 0.42 (0.18,1.8)	^a^ ^,^ ^c^<0.00^b^,^a^ ^,b^0.21	0.66 ± 0.32 (0.39,0.7)	0.80 ± 0.35 (0.2,1.7)	0.84 ± 0.39 (0.2,1.9)	^a^ ^,a^0.87,^a^ ^,b^0.76
ALP (U/L)	131.22 ± 62.4 (72,291)	114.72 ± 12.34 (101,129)	^e,^<0.00^b^	154.93 ± 51.15 (89,290)	115.94 ± 12.08 (101,129)	114.84 ± 12.38 (97,131)	^a^ ^,^ ^c^<0.00^b^,^a^ ^,b^0.91	106.24 ± 8.41 (100,121)	109.98 ± 20.94 (50,129)	116.52 ± 12.19 (106,149)	^a^ ^,a^0.87,^a^ ^,b^0.91
Bilirubin (mg/dL)	0.63 ± 0.3 (0.23,1.70)	0.66 ± 0.12 (0.49,0.87)	^e,^0.99	0.72 ± 0.32 (0.3,1.6)	0.65 ± 0.11 (0.56,0.80)	0.69 ± 0.13 (0.58,0.97)	^a^ ^,a^1.00, 1.00	0.76 ± 0.11 (0.56,0.87)	0.65 ± 0.11 (0.56,0.8)	0.65 ± 0.12 (0.51,0.86)	^a^ ^,a^0.81,^a^ ^,b^1.00
SGPT (U/L)	45.08 ± 21.42 (18,97)	38.90 ± 8.89 (18,66)	^e,^<0.00^b^	36.86 ± 8.42 (25,56)	35.47 ± 6.84 (18,58)	35.50 ± 6.68 (18,58)	^a^ ^,a^1.00,^a^ ^,b^1.00	40.00 ± 9.35 (38,90)	35.81 ± 8.95 (18,58)	36.16 ± 8.21 (20,62)	^a^ ^,a^0.99,^a^ ^,b^100
Hb (g/dL)	11.61 ± 1.80 (6,16.3)	13.53 ± 1 5 (11,17)	^e,^<0.00^b^	11.5 ± 2.13 (8,14)	12.21 ± 1.52 (8.60,16.10)	13.53 ± 2.13 (11,16.8)	^a^ ^,^ ^c^<0.00^b^,^a^ ^,b^0.41	11.88 ± 1.47 (8,15)	12.71 ± 1.37 (8.1,14.8)	13.80 ± 1.42 (12,16.6)	^a^ ^,^ ^c^<0.00^b^,^a^ ^,b^0.07
TLC (×10^9^/L)	13.40 ± 3.8 (6,29)	7.71 ± 1.6 (3.9,10.8)	^e,^<0.00^b^	13.53 ± 2.13 (8.34,18)	6.13 ± 1.74 (3.7,11.4)	7.61 ± 1.51 (4,9.8)	^a^ ^,^ ^c^<0.0^b^0,^a^ ^,b^0.73	14 ± 2.43 (9,18)	6.26 ± 2.24 (3.8,14.2)	7.04 ± 1.70 (3.9,10)	^a^ ^,^ ^c^<0.00^b^,^a^ ^,^ ^d^0.97
RBC (×10^12^/L	4.33 ± 0.53 (4,5.2)	4.38 ± 0.54 (4,5.13)	^e,^1.00	4.2 ± 0.54 (3.7,5.9)	4.27 ± 0.66 (3.2,5.9	4.33 ± 0.54 (4.,5.12)	^a^ ^,a^0.99,^a^ ^,b^1.00	4.26 ± 0.53 (3.6,5.1)	4.28 ± 0.60 (3.1,5.3)	4.36 ± 0.56 (4,5.2)	^a^ ^,a^1.00,^a^ ^,b^1.00
Platelet (×10^9^/L)	161.43 ± 30 (109,297)	286.38 ± 36.5 (156,386)	^e,^<0.00^b^	154.6 ± 43.88 (112,299)	230.94 ± 64.22 (127,398)	276 ± 30 (139,367)	^a^ ^,^ ^c^<0.00^b^,^a^ ^,b^0.09	168.3 ± 49.2 (119,290)	223.03 ± 61.15 (118,399)	277.7 ± 45.56 (156,357)	^a^ ^,^ ^c^<0.00^b^,^a^ ^,b^0.06
Lym (%)	9.44 ± 2.95 (5,16)	32.80 ± 3.67 (22,38)	^e,^<0.00^b^	13.06 ± 3.80 (8,20)	25.51 ± 5.22 (17,44)	33 ± 3.60 (24,38)	^a^ ^,^ ^c^<0.00^b^,^a^ ^,b^0.06	12.94 ± 2.50 (11,17)	28.10 ± 6.81 (16,47)	32.3 ± 3.8 (21,40)	^a^ ^,^ ^c^<0.00^b^,^a^ ^,b^0.07
Mon (%)	3.14 ± 1.77 (1,9)	4.81 ± 1.43 (2,7)	^e,^<0.00^b^	3.21 ± 2.15 (2,10)	3.52 ± 1.24 (1,6)	4.85 ± 1.42 (2,7)	^a^ ^,^ ^c^<0.00^b^,^a^ ^,b^0.01^b^	1.59 ± 0.50 (1,2)	3.68 ± 1.37 (1,5)	4.36 ± 1.41 (2,7)	^a^ ^,^ ^c^<0.00^b^,^a^ ^,b^0.09
Neu (%)	86.67 ± 3.56 (80,92)	58.29 ± 3.26 (53,66)	^e,^<0.00^b^	82.31 ± 3.29 (77,88)	65.8 ± 5.73 (48,76)	58.23 ± 2.75 (52,62)	^a^ ^,^ ^c^<0.00^b^,^a^ ^,b^0.03^b^	83.12 ± 2.26 (81,88)	64.68 ± 7.70 (42,79)	59.2 ± 2.8 (8,66)	^a^ ^,^ ^c^<0.00^b^,^a^ ^,b^0.06
Bas (%)	0.19 ± 0.58 (0.0,2)	1.42 ± 0.49 (1,2)	^e,^<0.00^b^	0.06 ± 0.26 (0.0,2)	1.85 ± 0.83 (1,4)	1.58 ± 0.85 (1,4)	^a^ ^,^ ^c^<0.09^b^,^a^ ^,^0.08	0.18 ± 0.38 (0.0,1)	1.98 ± 0.96 (0,4)	1.34 ± 0.68 (1,5)	^a^ ^,^ ^c^<0.00^b^,^a^ ^,b^0.04
Eos (%)	1.36 ± 1.15 (0.0,5)	2.54 ± 0.71 (1,4)	^e,^<0.00^b^	1.43 ± 0.82 (1,4)	3.31 ± 1.13 (0,5)	2.52 ± 0.73 (1,4)	^a^ ^,^ ^c^<0.00^b^,^a^ ^,b^0.07	0.59 ± 0.50 (0.0,1)	2.56 ± 1.37 (0,6)	2.68 ± 0.62 (1,4)	^a^ ^,^ ^c^<0.00^b^,^a^ ^,b^0.98
NLR	10.19 ± 3.46	1.84 ± 0.39	^e,^<0.00^b^	6.91 ± 2.13	2.7 ± 0.7 (1.09,4.35)	1.44 ± 0.3	^a^ ^,^ ^c^<0.00^b^,^a^ ^,b^0.06	6.67 ± 1.29	2.53 ± 0.89 (0.89,4.93)	1.88 ± 0.36	^a^ ^,^ ^c^ <0.00^b^,^a^ ^,b^0.06
NMR	37.87 ± 21.63	13.77 ± 6.03	^e,^<0.00^b^	31.97 ± 10.74	22.31 ± 13.46 (10.33,76)	13.00 ± 5.87	^a^ ^,^ ^c^<0.00^b^,^a^ ^,b^0.00^b^	59 ± 21.74	33.49 ± 21.56 (8.4,79)	15.57 ± 6.78	^a^ ^,^ ^c^<0.00^b^,^a^ ^,b^0.00^b^

^a^ ANOVA, followed by Tukey’s multiple comparison test.

^b^ *p*-value was significant when less than 0.05. Data is presented as mean ± SD.

^c^ Disease with COVID-19, vs. Control.

^d^ Disease without COVID-19, vs. Control.

^e^ Student t-test.

**FIGURE 3:**
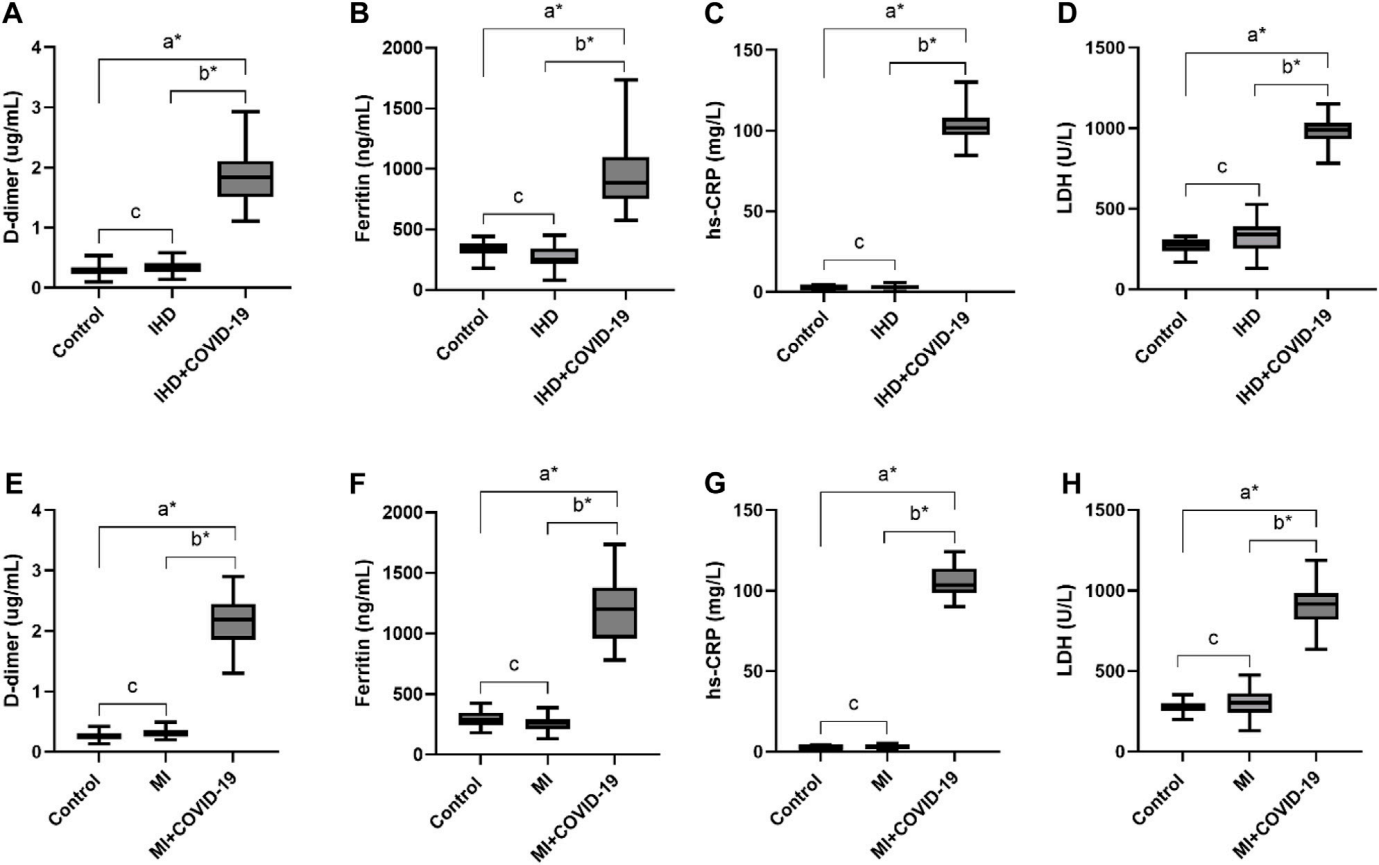
Elevated levels of D-dimer, Ferritin, hs-CRP and LDH in IHD and MI patients infected with COVID-19. **(A)**, D-dimer. **(B)**, Ferritin, **(C)**, hs-CRP and **(D)**, LDH levels in IHD patients suffered from COVID-19 infection. **(E)**, D-dimer. **(F)**, Ferritin, **(G)**, hs-CRP and **(H)**, LDH levels in MI patients infected with COVID-19.δ *p value was significant when less than 0.05 ^a^ Disease with COVID-19 vs healthy control ^b^ Disease with COVID-19 vs disease control ^c^ Disease without COVID-19 vs healthy control.

D-dimer levels were also significantly high in the IHD and MI group infected with COVID-19 (1.85 ± 0.48 and 2.02 ± 0.5 μg/mL, respectively). In addition, serum ferritin levels were also significantly high in the present disease group (918.47 ± 263.13 and 1,203.59 ± 336.43 μg/L). The overproduction of inflammatory cytokines in COVID-19 patients with severe disease may cause high CRP values. Herein, the hs-CRP level was also significantly high in the severe disease group of COVID-19, with 102.22 ± 7.62 and 104.55 ± 9.31 mg/L. However, the level of LDH was also high in the respective disease group of COVID-19 patients (998.53 ± 101.34, 896.35 ± 126.8 U/L) shown in Table 2 and Figures 3A–H.

**TABLE 2:** Clinical Characteristics of IHD and MI patients infected with COVID-19.

	IHD + COVID-19	IHD	Control	*p-*value	MI + COVID-19	MI	Control	*p-*value
M/F	40/20	44/28	36/20		24/44	30/43	19/24	
M/F (%)	66.7/33.3	61/39	64.3/35.7		35.3/64.7	41/59	44.2/55.8	
Age (Year)	61.41 ± 10.8 (34,82)	60.66 ± 11.88 (33,80)	64.26 ± 10.83 (33,80)	^a^0.63,^b^0.45	63.36 ± 13.48 (30,85)	58.94 ± 17.97 (22,80)	65.51 ± 16.66 (27,79)	^a^0.37,^b^0.12
Systolic (mmHg)	140.33 ± 4.99 (130,145)	122.37 ± 11.02 (103,155)	120.18 ± 8.79 (105,135)	^a^<0.00,^b^0.97	131.76 ± 8.45 (134,110)	118.24 ± 11.09 (80,135)	119.19 ± 9.32 (105,135)	^a^<0.00,^b^0.98
Diastolic (mmHg)	91.40 ± 9.15 (79,110)	78.59 ± 6.21 (70,95)	79.55 ± 6.62 (70,90)	^a^<0.00,^b^0.96	84.12 ± 6.04 (75,95)	78.45 ± 5.46 (70,90)	80.12 ± 6.12 (70,90)	^a^0.97,^b^0.78
D Dimer (µg/mL)	1.85 ± 0.48 (1.30,2.90)	0.33 ± 0.11 (0.14,0.57)	0.28 ± 0.09 (0.14,0.41)	^a^<0.00^b^,^b^0.09	2.02 ± 0.5 (1.3,2.9)	0.32 ± 0.07 (0.2,0.49)	0.27 ± 0.08 (0.13,0.41)	^a^<0.00^b^,^b^0.08
Ferritin (ng/mL)	918.47 ± 263.13 (681,1737)	247.63 ± 89.62 (78,430)	334.23 ± 82.05 (178,423)	^a^<0.00^b^,^b^0.09	1,203.59 ± 336.43 (781,1737)	266.38 ± 62.73 (130,407)	298.42 ± 79.6 (180,422)	^a^<0.00^b^,^b^0.89
RBS (mg/dL)	150.80 ± 25.53 (104.00,195)	162.23 ± 25.36 (120,198)	162.21 ± 25.23 (118,194)	^a^0.99,^b^1.00	148.06 ± 25.32 (102,189)	157.99 ± 27.30 (120, 205)	159.19 ± 26.57 (116,198)	^a^0.99,^b^1.00
FBS (mg/dL)	88 ± 9.69 (76,98)	87.72 ± 9.75 (67,107)	89.16 ± 7.38 (77,100)	^a^1.00,^b^0.98	87.12 ± 7.8 5 (71,101)	89.51 ± 7.74 (78,110)	88.65 ± 6.7 (72,105)	^a^1.00,^b^0.99
HbA1c (%)	3.96 ± 0.47 (3.8,5.5)	4.82 ± 0.62 (3.80,5.60)	4.77 ± 0.64 (3.90,5.3)	^a^0.97,^b^0.99	5.09 ± 0.65 (4,7)	4.75 ± 0.59 (3.5,5.60)	4.83 ± 0.55 (3.6,5.3)	^a^0.98,^b^1.00
Blood Urea (mg/dL)	37.67 ± 25.97 (21,103)	41.8 ± 20.7 8 (21,70)	39.73 ± 20.78 (21.00,65)	^a^1.00,^b^0.99	65.38 ± 44.34 (23,300)	36.62 ± 19.57 (21,70)	37.44 ± 20 (22,78)	^a^<0.00^b^,^b^0.98
CRP mg/L	102.22 ± 7.62 (92,118)	2.8061111 ± 1.47 (0.20,5.30)	2.84 ± 0.71 (1.4,4.5)	^a^<0.00^b^,^b^1.00	104.55 ± 9.31 (90,124)	3.14 ± 0.81 (1.4,4.90)	2.69 ± 0.62 (1.8,3.9)	^a^<0.00^b^,^b^0.94
S LDH U/L	998.53 ± 101.34 (781,1112)	319.68 ± 97.92 (129,493)	264.73 ± 43.32 (190,316)	^a^<0.00^b^,^b^0.67	896.35 ± 126.8 (434,1205)	304.48 ± 82.61 (128,476)	275.84 ± 33.29 (201,352)	^a^<0.00^b^,^b^0.56
Creatinine (mg/dL)	0.92 ± 0.62 (0.39,2.1)	0.85 ± 0.41 (0.20,1.70)	0.85 ± 0.41 (0.19,1.9)	^a^1.00,^b^1.00	2.27 ± 1.45 (0.8,6.9)	0.92 ± 0.46 (0.2,1.90)	0.89 ± 0.45 (0.27,1.81)	^a^<0.00^b^,^b^0.98
ALP (U/L)	111.8 ± 13.74 (82.00,137)	113.94 ± 12.08 (101,129)	115.93 ± 12.1 (105,131)	^a^1.00,^b^1.00	127.41 ± 53.42 (72,292)	109.46 ± 17.16 (68,129)	114.67 ± 12.13 (104,123)	^a^0.98,^b^0.99
Bilirubin (mg/dL)	0.71 ± 0.14 (0.40,0.8)	0.65 ± 0.11 (0.56,0.80)	0.61 ± 0.12 (0.58,0.79)	^a^0.98,^b^0.81	0.70 ± 0.41 (0.3,1.7)	0.79 ± 0.14 (0.52,1)	0.68 ± 0.14 (0.55,1)	^a^0.97,^b^1.00
SGPT (U/L)	36.73 ± 13.6 (20,85)	35.47 ± 6.84 (18,74)	35.8 ± 6.98 (19,58)	^a^1.00,^b^1.00	47.82 ± 23.45 (20,97)	40.25 ± 9.35 (18,60)	38.23 ± 9.89 (21,61)	^a^0.10,^b^0.81
Hb (g/dL)	10.83 ± 1.85 (7,14)	12.21 ± 1.52 (8.60,16.10)	13.8 ± 1.48 (11,16.7)	^a^<0.00^b^,^b^0.08	11.441.68 (7.8,14)	12.10 ± 1.39 (8.9,15.10)	14.1 ± 1.25 (12.4,17)	^a^<0.00^b^,^b^0.03
TLC (×10^9^/L)	14.3 ± 3 (9,20)	6.13 ± 1.74 (3.7,11.4)	7.39 ± 2 (3.8,10.7)	^a^<0.00^b^,^b^0.78	13.38 ± 3.06 (11,23)	6.10 ± 1.95 (3.6,13.5)	7.45 ± 1.80 (4,11.6)	^a^<0.00^b^,^b^0.34
RBC (×10^12^/L	4 ± 0.51 (3.2,5.7)	4.27 ± 0.66 (3.2,5.9)	4.39 ± 0.57 (3.9,5.2)	^a^0.21,^b^0.89	4.34 ± 0.76 (3.2,5.7)	4.91 ± 5.58 (3.1,51)	4.39 ± 0.57 (4,5.2)	^a^1.00,^b^0.89
Platelet (×10^9^/L)	173.55 ± 41.00 (105,290)	230.94 ± 64.22 (127,398)	271 ± 31.7 (148,310)	^a^<0.00^b^,^b^0.08	171.1 ± 50.5 (98,298)	232.39 ± 61.84 (126,399)	283.25 ± 35.33 (98,298)	^a^<0.00^b^,^b^0.13
Lym (%)	13.27 ± 4.40 (5,17)	25.51 ± 5.22 (17,44)	32.7 ± 3.4 (23,37)	^a^<0.00^b^,^b^0.15	9.24 ± 2.98 (5,16)	28.44 ± 6.52 (18,44)	33.49 ± 3.54 (20,38)	^a^<0.00^b^,^b^0.34
Mon (%)	2.07 ± 0.77 (1.00,4)	3.52 ± 1.24 (1,6)	4.73 ± 1.45 (2,7)	^a^<0.00^b^,^b^0.06	3 ± 1.82 (1,9)	2.80 ± 1.21 (1,6)	4.98 ± 1.28 (2,7)	^a^<0.00^b^,^c^<0.00^b^
Neu (%)	83.07 ± 4.04 (79,92)	65.8 ± 5.73 (48,76)	58.58 ± 3.12 (49,68)	^a^<0.00^b^,^b^0.08	87.12 ± 3.33 (80,92)	63.77 ± 7.38 (48,77)	57.32 ± 3.8 (51,66)	^a^<0.00^b^, 0.07
Bas (%)	0.67 ± 0.7 (0.0,2)	1.85 ± 0.83 (1,4)	1.34 ± 0.48 (1,5)	^a^<0.00^b^,^b^0.91	0.12 ± 0.47 (0.0,2)	1.94 ± 1.12 (0,4)	1.67 ± 0.79 (1,3)	^a^<0.00^b^,^b^0.07
Eos (%)	0.93 ± 0.44 (0.0,2)	3.31 ± 1.13 (0,5)	2.61 ± 0.65 (1,4)	^a^<0.00^b^,^b^0.02	1.35 ± 1.19 (0.0,5)	3.04 ± 1.62 (0,6)	2.44 ± 0.73 (1,4)	^a^<0.00^b^,^b^0.08
NLR	7.61 ± 4.19	2.7 ± 0.7 (1.09,4.35)	1.84 ± 0.32	^a^<0.00^b^,^b^0.02	10.43 ± 3.4	2.41 ± 0.76 (1.11,4.11)	1.77 ± 0.37	^a^<0.00^b^,^b^0.05^b^
NMR	46.50 ± 19.47	22.31 ± 13.46 (10.33,76)	14.16 ± 6.41	^a^<0.00^b^,^c^<0.00^b^	41.23 ± 23.8	28.06 ± 15.26 (10,74)	12.66 ± 5.04	^a^<0.00^b^,^c^<0.00^b^

δ ANOVA, followed by Tukey’s multiple comparison test.

^a^ Disease with COVID-19 vs. Control.

^b^ *p*-value was significant when less than 0.05. Data is presented as mean ± SD.

^c^ Disease without COVID-19, vs. Control.

**FIGURE 4:**
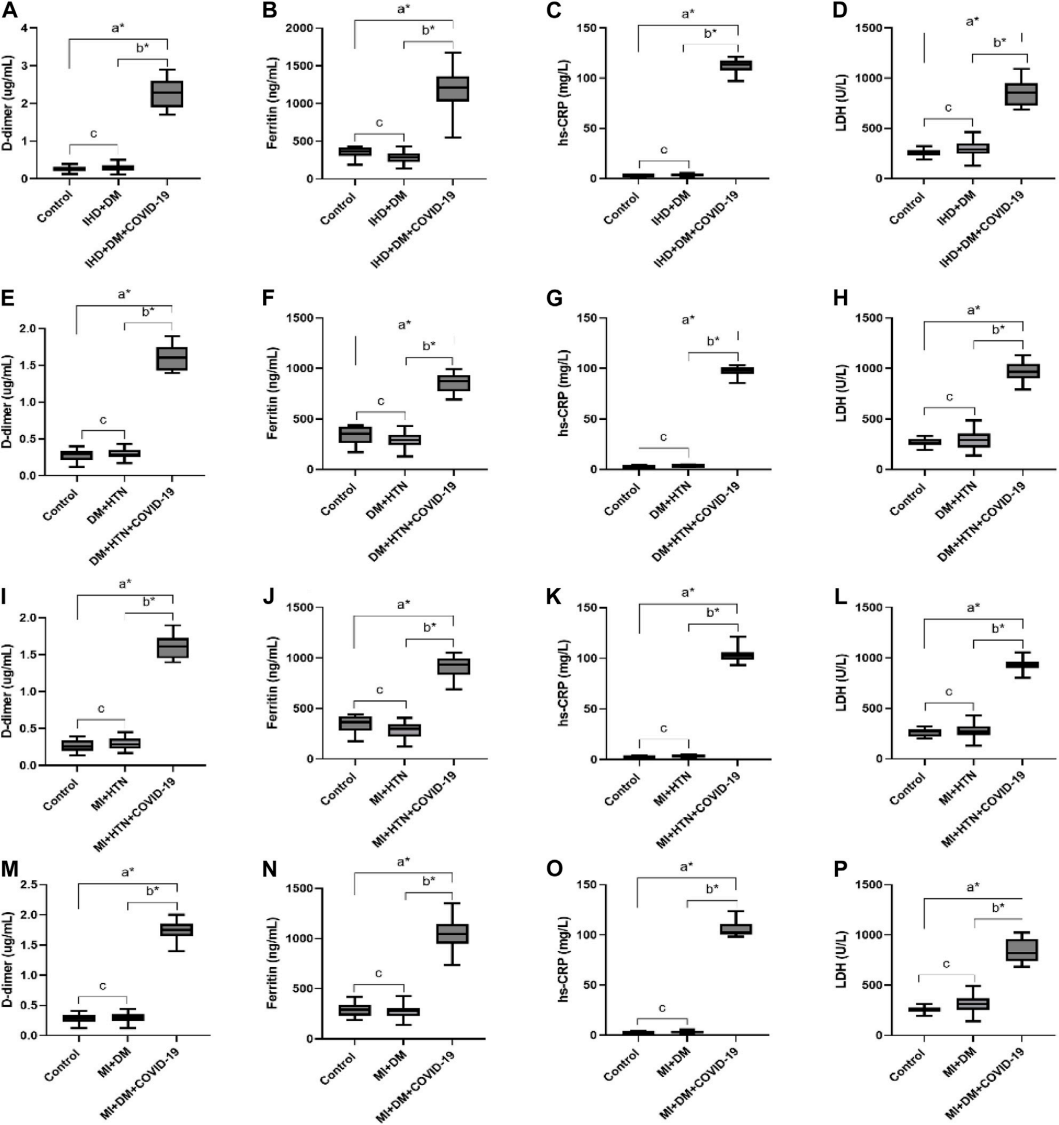
Increased levels of D-dimer, Ferritin, hs-CRP and LDH in different disease conditions of IHD with DM, DM with HTN, MI with HTN and MI with DM patients infected with COVID-19. **(A)**, D-dimer. **(B)**, Ferritin, **(C)**, hs-CRP and **(D)**, LDH levels in IHD with DM patients suffered from COVID-19 infection. **(E)**, D-dimer. **(F)**, Ferritin, **(G)**, hs-CRP and **(H)**, LDH levels in DM with HTN patients infected with COVID-19. **(I)**, D-dimer. **(J)**, Ferritin, **(K)**, hs-CRP and **(L)**, LDH levels in MI with HTN patients suffered from COVID-19 infection. **(M)**, D-dimer. **(N)**, Ferritin, **(O)**, hs-CRP and **(P)**, LDH levels in MI with DM patients suffered from COVID-19 infection.δ *p value was significant when less than 0.05 ^a^ Disease with COVID-19 vs healthy control ^b^ Disease with COVID-19 vs disease control ^c^ Disease without COVID-19 vs healthy control.

Previous studies have shown that D-dimer and fibrinogen concentrations increase in the early stages of COVID-19 disease. In the present cohort, patients of the multiple disease groups IHD with DM, DM with HTN, MI with HTN, and MI with DM infected with COVID-19 also showed significantly elevated levels of D-dimers (2.13 ± 0.50, 1.56 ± 0.21, 1.58 ± 0.20 and 1.77 ± 0.19 μg/mL). Furthermore, serum ferritin levels were also significantly high in disease present disease group of COVID-19 with high values of 1,177.7 ± 284.95, 848.58 ± 116.54, 913.50 ± 110.61, and 1,046.08 ± 181.30 μg/L, respectively. The hs-CRP level was also significantly high in the disease group of COVID-19 (112.14 ± 5.88, 98.29 ± 4.48, 102.33 ± 4.45, 105.00 ± 8.87 mg/L, respectively). In the cells of the majority of body tissues, lactate is converted to pyruvate by an enzyme called lactate dehydrogenase (LDH), and its activity is elevated after tissue breakdown. However, the LDH concentration was also significantly high in the group of COVID-19 patients with respective disease conditions (858.64 ± 155.87, 982.25 ± 103.27, 934.75 ± 54.73, 817.50 ± 131.05 U/L respectively) given in Table 3 and Figures 4A–P.

**TABLE 3:** Clinical characteristics of IHD + DM, DM + HTN, MI + HTN and MI + DM patients infected with COVID-19.

	IHD + DM + COVID-19	IHD + DM	Control	*p-*value	DM + HTN + COVID-19	DM + HTN	Control	*p-*value	MI + HTN + COVID-19	MI + HTN	Control	*p-*value	MI + DM + COVID-19	MI + DM	Control	*p-*value
M/F	32/24	17/35	21/20		28/20	24/20	21/17		20/27	20/24	15/17		16/32	18/34	13/27	
M/F (%)	57.1/42.9	32/68	51.2.48.8		58.4/41/6	55/45	51.2/48.8		42.6/57.4	45/55	46.9/53.1		33.4/66.6	35/65	32.5/67.5	
Age (Year)	58.30 ± 12.67 (33,80)	61.58 ± 18.16 (24,82)	62.80 ± 13.26 (37,80)	^a^0.45,^b^0.98	64.14 ± 14.10 (33,81)	60.45 ± 16.70 (24,82)	65 ± 16 (28,87)	^a^0.7,^b^0.07	69.29 ± 13.98 (30,82)	62.45 ± 17.66 (27,84)	65.84 ± 15.70 (31,83)	^a^0.4,^b^0.91	65.58 ± 13.64 (34,80)	61.58 ± 18.16 (24,82)	72.80 ± 9.37 (25,82)	^a^0.00^a^,^b^0.09
Systolic (mmHg)	131.79 ± 9.65 (110,145)	117.88 ± 9.64 (100,135)	120.12 ± 8.18 (105,135)	^b^<0.00^a^,^b^0.56	187.67 ± 16.58 (160,210)	191.63 ± 13.79 (160,210)	120.60 ± 7.93 (105,135)	^b^<0.00^a^,^c^<0.00^a^	195.92 ± 10.03 (180,210)	193.46 ± 13.23 (160,210)	120.31 ± 8.22 (105,135)	^b^<0.00^a^,^c^<0.00^a^	134.17 ± 10.88 (110,150)	117.88 ± 9.64 (100,135)	119.50 ± 9.66 (105,135)	^a^0.00^a^,^b^0.89
Diastolic (mmHg)	86.79 ± 5.91 (80,95)	78.77 ± 5.52 (70,95.00)	79.63 ± 6.46 (70,90)	^b^<0.00^a^,^b^0.54	105.00 ± 4.46 (95,110)	100.90 ± 7.79 (90,110)	77.81 ± 5.12 (70,85)	^a^0.00^a^ 0.00^a^	99.083.29 (95,105)	100.27 ± 8.07 (90,110)	79.53 ± 7 (70,90)	^a^0.00^a^,^b^0.00^a^	87.50 ± 6.68 (80,95)	78.77 ± 5.52 (70,95)	80.38 ± 6.24 (70,90)	^a^0.00^a^,^b^0.03^a^
D Dimer (µg/mL)	2.13 ± 0.50 (1.70,2.90)	0.29 ± 0.09 (0.12,0.50)	0.27 ± 0.08 (0.12,0.39)	^b^<0.00^a^,^b^0.56	1.56 ± 0.21 (1.40,1.90)	0.30 ± 0.07 (0.17,0.43)	0.27 ± 0.08 (0.12,0.40)	^a^0.00^a^,^b^0.32	1.58 ± 0.20 (1.40,1.90)	0.29 ± 0.08 (0.15,0.45)	0.27 ± 0.09 (0.14,0.39)	^a^0.00^a^,^b^0.78	1.77 ± 0.19 (1.40,2)	0.29 ± 0.09 (0.12,0.50)	0.27 ± 0.08 (0.12,0.41)	^a^0.00^a^,^b^0.78
Ferritin (ng/mL)	1,177.7 ± 284.95 (403,1672)	375.75 ± 64.92 (128,431)	352.32 ± 74.20 (170,430)	^b^<0.00,^b^0.35	848.58 ± 116.54 (691,995)	298.25 ± 93.38 (129,431)	357.08 ± 74.62 (170,434)	^a^0.00^a^,^b^0.19	913.50 ± 110.61 (690,1001)	285.97 ± 75.69 (123,406)	348.41 ± 82.02 (178,440)	^a^0.00^a^,^b^0.07	1,046.08 ± 181.30 (681,1320)	275.75 ± 64.92 (128, 431)	294.13 ± 82.60 (190,422)	^a^0.00^a^,^b^0.46
RBS (mg/dL)	421.21 ± 87.21 (301,530)	441.92 ± 67.47 (319,561.00)	162.07 ± 25.63 (113,191)	^b^<0.00^a^,^c^<0.00^a^	413.42 ± 99.64 (240,560)	433.86 ± 75.84 (319,561)	163.64 ± 24.77 (121,182)	^b^<0.00^a^,^c^<0.00^a^	175.75 ± 18.50 (151,200)	153.11 ± 29.53 (104,198)	164.13 ± 25.19 (123,190)	^a^1.00,^b^0.98	473.83 ± 58.67 (397,581)	441.92 ± 67.47 (319,561)	160.43 ± 27.13 (121,197)	^b^<0.0^a^0,^cc^<0.00^a^
FBS (mg/dL)	193.36 ± 10.22 (181,214)	199.58 ± 7.13 (189,209.00)	89.27 ± 7.38 (78,104)	^b^<0.00^a^,^c^<0.00^a^	178.17 ± 39.85 (120,278)	197.25 ± 18.17 (140,260)	89.46 ± 7.65 (77,101)	^b^<0.00^a^,^c^<0.00^a^	93.17 ± 8.81 (79,103)	89.68 ± 11.27 (67,120)	90.16 ± 7.71 (74,102)	^a^0.98,^b^1.00	192.50 ± 8.82 (170,201)	199.58 ± 7.13 (189,209)	89.30 ± 6.71 (77,105)	^a^0.00^a^,^c^<0.00^a^
HbA1c (%)	10 ± 1.56 (8,13)	10.09 ± 1.89 (7,14.10)	4.66 ± 0.71 (3.70,5.80)	^b^<0.00^a^,^c^<0.00^a^	9.80 ± 1.66 (7,13)	10.65 ± 2.16 (7,14.1)	4.69 ± 0.71 (3.20,5)	^a^0.00^a^,^b^0.00^a^	4.22 ± 0.38 (3.90,5)	4.52 ± 0.64 (3.3,5.6)	4.82 ± 0.67 (3.90,5.70)	^a^0.58,^b^0.84	9.50 ± 1.00 (7,12)	10.09 ± 1.89 (7,14.10)	4.91 ± 0.51 (3.20,5.50)	^a^0.00^a^,^c^<0.00^a^
Blood Urea (mg/dL)	61.57 ± 41.97 (23,120)	34.69 ± 19.23 (21,70.00)	41.24 ± 21.53 (21,63)	^a^0.29,^b^0.85	49.08 ± 17.68 (22,65)	39.27 ± 20.93 (20,70)	41.26 ± 21.41 (25,74)	^a^1.00,^b^0.97	45.67 ± 17.10 (20,65)	36.89 ± 19.13 (20,74)	41.22 ± 21.72 (26,72)	^a^1.00,^b^0.97	83.75 ± 34.51 (23,120)	34.69 ± 19.23 (21,70)	38.08 ± 20.55 (19,64)	^a^0.00^a^,^b^1.00
CRP mg/L	112.14 ± 5.88 (97,121)	3.50 ± 0.93 (1.7,5.70)	2.86 ± 0.72 (1.70,4.20)	^b^<0.00^a^,^b^0.41	98.29 ± 4.48 (77,103)	3.52 ± 0.86 (2.1,5.3)	2.89 ± 0.72 (1.60,4.20)	^a^0.00^a^,^b^0.51	102.33 ± 4.45 (90,121)	3.23 ± 1.09 (1.4,5.20)	2.83 ± 0.71 (2.10,3.80)	^a^0.00^a^,^b^0.31	105.00 ± 8.87 (98,125)	3.50 ± 0.93 (1.7,5.70)	2.68 ± 0.61 (1.90,4.30)	^a^0.00^a^,^b^0.32
S LDH U/L	858.64 ± 155.87 (686,1092)	296.17 ± 86.31 (126,492)	264.37 ± 43.21 (190,320)	^b^<0.00^a^,^b^0.54	982.25 ± 103.27 (890,1129)	315.20 ± 87.97 (139,490)	262.86 ± 45.01 (190,330)	^a^0.00^a^,^b^0.64	934.75 ± 54.73 (898,1130)	27.95 ± 78.56 (129,429)	262.16 ± 45.94 (201,318)	^a^0.00^a^,^b^0.89	817.50 ± 131.05 (686,1023)	296.17 ± 86.31 (126,492)	271.90 ± 38.23 (190,314)	^a^0.00^a^,^b^0.67
Creatinine (mg/dL)	1.66 ± 0.98 (0.40,3.10)	0.91 ± 0.44 (0.2,1.70)	0.86 ± 0.39 (0.21,1.90)	^b^<0.00^a^,^b^0.99	2.23 ± 0.60 (1.30,3.10)	0.89 ± 0.41 (0.2,1.7)	0.84 ± 0.39 (0.16,1.67)	^a^0.00^a^,^b^0.78	0.73 ± 0.35 (0.39,1.90)	0.81 ± 0.36 (0.2,1.70)	0.84 ± 0.39 (0.18,1.80)	^a^1.00,^b^0.99	2.37 ± 0.46 (1.80,3.20)	0.91 ± 0.44 (0.2, 1.70)	0.89 ± 0.44 (0.19,1.70)	^a^0.00^a^,^b^0.78
ALP (U/L)	141.00 ± 53.98 (90,232)	107.10 ± 18.51 (47,129.00)	116.41 ± 12.28 (93,141)	^a^0.30,^b^0.25	119.33 ± 17.33 (100,170)	108.89 ± 23.95 (40,129)	116.52 ± 12.19 (95.00,139.00)	^a^1.00,^b^0.99	128.50 ± 16.03 (101,159)	106.19 ± 25.27 (40,137)	116.25 ± 12.42 (98,133)	^a^1.00,^b^0.97	176.17 ± 43.98 (101,232)	107.10 ± 18.51 (47,129)	114.70 ± 12.32 (103,127)	^a^0.00^a^,^b^0.04
Bilirubin (mg/dL)	1.02 ± 0.51 (0.46,2)	0.76 ± 0.14 (0.56,1)	0.65 ± 0.12 (0.58,0.83)	^b^<0.00^a^,^b^0.98	0.73 ± 0.13 (0.50,0.92)	0.75 ± 0.14 (0.56,1)	0.65 ± 0.12 (0.49,1)	^a^1.00,^b^1.00	0.75 ± 0.14 (0.52,0.89)	0.72 ± 0.16 (0.5,1)	0.65 ± 0.12 (0.50,0.81)	^a^0.98,^b^0.76	1.24 ± 0.52 (0.57,1.98)	0.76 ± 0.14 (0.56,1)	0.76 ± 0.14 (0.50,0.98)	^a^0.00^a^,^b^1.00
SGPT (U/L)	56.57 ± 16.24 (38,90)	35.69 ± 8.93 (18,58.00)	36.60 ± 7.98 (20,68)	^b^<0.00^a^,^b^0.98	39.75 ± 9.88 (32,60)	36.27 ± 6.88 (20,50)	37.11 ± 5.64 (30,50)	^a^1.00,^b^1.00	41.50 ± 11.22 (27,68)	33.54 ± 10.03 (18,50)	36.25 ± 10.06 (20,56)	^a^0.99,^b^1.00	61.42 ± 21.76 (34,90)	35.69 ± 8.93 (18, 58)	35.70 ± 9.07 (17,60)	^a^0.00^a^,^b^1.00
Hb (g/dL)	11.00 ± 1.34 (8,13)	13.97 ± 1.42 (8.80,15.20)	14.17 ± 1.26 (11.70,17)	^b^<0.00^a^,^b^0.43	11.69 ± 1.35 (7.8,12.9)	12.00 ± 1.59 (8.8,15.2)	13.1 ± 1.34 (8.4,16.3)	^a^0.00^a^,^b^0.78	11.16 ± 1.40 (8,13)	13.37 ± 1.57 (9.60,16.10)	14.03 ± 1.14 (12,17)	^a^0.00^a^,^b^0.45	11.83 ± 1.53 (9,14)	13.97 ± 1.42 (8.80, 15.20)	14.12 ± 1.24 (12,17)	^a^0.00^a^,^b^0.08
TLC (×10^9^/L)	13.37 ± 2.00 (9.20,18)	6.46 ± 2.35 (3.2,14.10)	7.07 ± 1.73 (4.,10)	^b^<0.00^a^,^b^0.76	13.02 ± 2.08 (10,20)	6.13 ± 2.26 (3.1,14.2)	7.23 ± 1.80 (3.80,10.70)	^a^0.00^a^,^b^0.87	13.35 ± 2.33 (10,19.70)	6.39 ± 2.58 (3.9,15.10)	7.21 ± 2 (4,11)	^a^0.00^a^,^b^0.87	13.25 ± 1.90 (10,18)	7.86 ± 2.35 (6.2,14.10)	8.17 ± 1.48 (5,11)	^a^0.00^a^,^b^0.67
RBC (×10^12^/L	4.12 ± 0.62 (3.90,5.50)	3.88 ± 0.55 (3.1,5.20)	4.35 ± 0.55 (4.00,5.11)	^a^1.00,^b^1.00	4.37 ± 0.59 (3.60,5.10)	4.23 ± 0.52 (3.1,5.2)	4.36 ± 0.56 (3.90,5.30)	^a^1.00,^b^1.00	4.38 ± 0.67 (3.60,5.20)	4.34 ± 0.51 (3.3,5.20)	4.45 ± 0.55 (4,5.13)	^a^1.00,^b^1.00	3.91 ± 0.74 (3.20,5.50)	4.32 ± 0.55 (3.1,5.20)	4.36 ± 0.56 (4,5.20)	^a^1.00,^b^1.00
Platelet (×10^9^/L)	160.10 ± 49.00 (115,278)	282.06 ± 102.50 (125,477)	282.30 ± 46.10 (187,370)	^b^<0.00,^b^1.00	177.80 ± 49.00 (129,290)	215.16 ± 54.80 (121,304)	271.10 ± 31.10 (159,310)	^a^0.00^a^,^b^0.74	161.70 ± 33.70 (121,278)	228.23 ± 62.42 (121,322)	263.50 ± 52 (145,370)	^a^0.00^a^,^b^0.73	175.10 ± 58.60 (109,198)	282.06 ± 102.50 (125, 477)	281.60 ± 38 (167,376)	^a^0.00^a^,^b^1.00
Lym (%)	13.50 ± 2.37 (8,20)	27.65 ± 8.21 (14,44.00)	32.76 ± 2.98 (23,37)	^b^<0.00^a^,^b^0.23	18.42 ± 19.48 (13.08,2.50)	28.45 ± 6.23 (20,44)	32.48 ± 3.90 (22,37)	^a^0.00^a^,^b^0.33	12.92 ± 1.96 (12.90,2)	29.59 ± 6.85 (15,45)	32.10 ± 4 (22,37)	^a^0.00^a^,^b^0.39	13.25 ± 2.45 (11,17)	27.65 ± 8.21 (14, 44)	32.80 ± 4.27 (21,39)	^a^0.00^a^,^b^0.45
Mon (%)	1.57 ± 0.50 (1,2)	3.31 ± 1.19 (1,6)	4.39 ± 1.39 (2,7)	^b^<0.00^a^,^b^0.56	1.17 ± 0.69 (0,2)	2.23 ± 1.26 (1,6)	4.36 ± 1.41 (2,7)	^a^0.00^a^,^b^0.00^a^	1.00 ± 0.58 (0,2)	3.91 ± 1.29 (1,6)	4.50 ± 1.50 (2,7)	^a^0.00^a^,^b^0.74	0.92 ± 0.50 (0,2)	3.31 ± 1.19 (1,6)	4.05 ± 1.32 (2,6)	^a^0.00^a^,^b^0.23
Neu (%)	83.50 ± 2.04 (81,86)	63.75 ± 8.71 (44,78.00)	58.74 ± 2.73 (50,65)	^b^<0.00^a^,^b^0.09	85.08 ± 2.42 (81,89)	65.41 ± 6.88 (46,76)	58.70 ± 3 (49,66)	^a^0.00^a^,^b^0.07	85.25 ± 2.15 (82,88)	62.27 ± 7.15 (46,80)	59.03 ± 3.61 (48,68)	^a^0.00^a^,^b^0.09	84.25 ± 2.65 (81,88)	63.75 ± 8.71 (44, 78)	57.93 ± 3.47 (58.22,4.26)	^a^0.00^a^,^b^0.07
Bas (%)	0.57 ± 0.50 (0,1)	2.00 ± 1.05 (0,4)	1.72 ± 0.74 (1,4)	^b^<0.00^a^,^b^0.05	0.25 ± 0.44 (0,1)	1.84 ± 1.10 (0,5)	1.34 ± 0.53 (1,3)	^a^0.00^a^,^b^009	0.25 ± 0.44 (0,1)	1.91 ± 0.94 (0,4)	1.46 ± 0.84 (1,5)	^a^0.00^a^,^b^0.84	0.50 ± 0.51 (0,1)	2.00 ± 1.05 (0,4)	1.37 ± 0.49 (1,2)	^a^0.00^a^,^b^0.08
Eos (%)	0.86 ± 0.64 (0,2)	3.29 ± 1.40 (0,6.00)	2.63 ± 0.62 (1,4)	^b^<0.00^a^,^b^0.76	0.58 ± 0.50 (0,1)	2.32 ± 1.33 (0,5)	2.68 ± 0.62 (1,4)	^a^0.00^a^,^b^0.84	0.58 ± 0.50 (0,1)	3.14 ± 1.30 (0,5)	2.63 ± 0.66 (1,4)	^a^0.00^a^,^b^0.08	1.08 ± 0.50 (0,2)	3.29 ± 1.40 (0, 6)	2.99 ± 0.75 (1,4)	^a^0.00^a^ ^b^0.23
NLR	6.40 ± 1.27	2.61 ± 1.14 (1,5.57)	2.13 ± 0.26	^a^0.00^a^,^b^0.76	6.77 ± 1.43	2.46 ± 0.70 (1.04,3.8)	2.44 ± 0.33	^a^0.00^a^,^b^0.87	6.76 ± 1.09	3.24 ± 0.92 (1.02,5.3)	2.68 ± 0.40	^a^0.00^a^,^b^0.29	6.59 ± 1.30	2.61 ± 1.14 (1,5.57)	1.83 ± 0.42	^a^0.00^a^,^b^0.08
NMR	59.50 ± 20.44	24.13 ± 17.10 (8.8,78.00)	15.27 ± 6.54	^b^<0/00^a^ ^c^<0/00^a^	70.96 ± 20.54	40.75 ± 21.80 (7.66,75)	15.23 ± 6.78	^b^<0/00^a^,^c^<0/00^a^	78.08 ± 16	17.00 ± 19.86 (9.83,80)	11.80 ± 7.21	^b^<0/00^a^,^c^<0/00^a^	80.67 ± 11.77	24.13 ± 17.10 (8.8, 78)	12.72 ± 5.15	^b^<0.00^a^,^c^<0/00^a^

δ ANOVA, followed by Tukey’s multiple comparison test.

^a^ *p*-value was significant when less than 0.05. Data is presented as mean ± SD.

^b^ Disease with COVID-19, vs. Control.

^c^ Disease without COVID-19, vs. Control.

**FIGURE 5:**
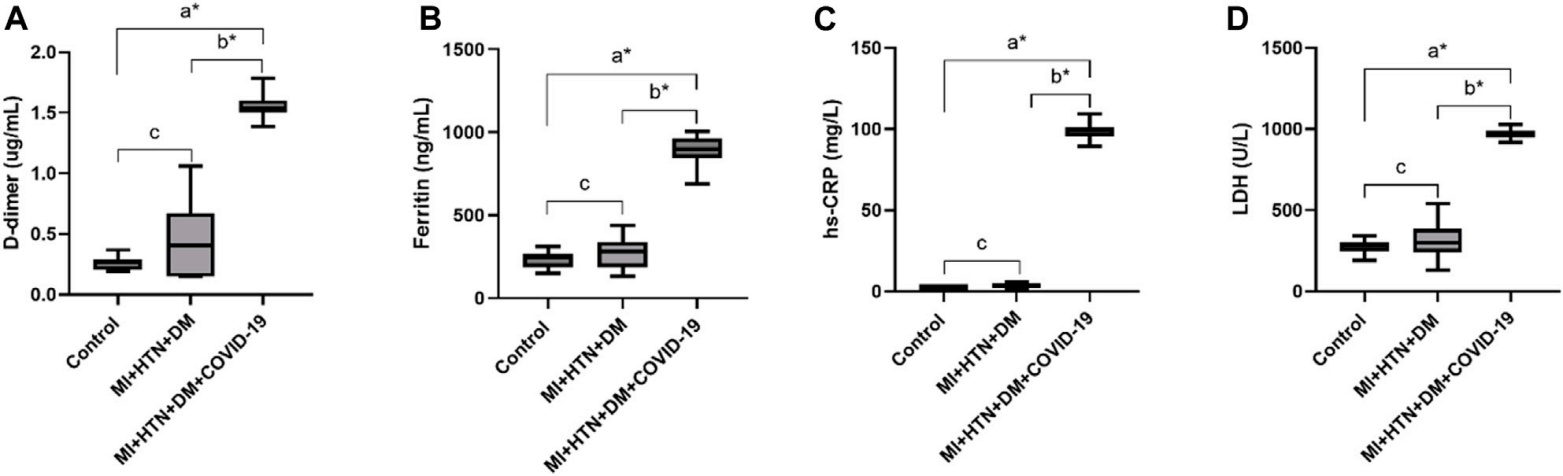
Increased levels of D-dimer, Ferritin, hs-CRP and LDH in disease condition of combine MI, HTN and DM patients infected with COVID-19. **(A)**, D-dimer. **(B)**, Ferritin, **(C)**, hs-CRP and **(D)**, LDH.δ *p value was significant when less than 0.05 ^a^ Disease with COVID-19 vs healthy control ^b^ Disease with COVID-19 vs disease control ^c^ Disease without COVID-19 vs healthy control.

The D-dimer level was also significantly high (1.54 ± 0.09 μg/mL) in the group of COVID-19 patients with multiple disease condition of MI, DM, and HTN. Furthermore, serum ferritin levels were also significantly high in the present disease group (861.43 ± 118.58) infected with COVID-19. The severe COVID-19 disease groups of MI, DM, and HTN also have significantly high levels of hs-CRP (87.00, 110.00 mg/L). In addition, the LDH level (970.29 ± 46.95 U/L) was also significantly raised in the present disease group of COVID-19 patients with MI, DM, and HTN (Table 4 and Figures 5A–D).

**TABLE 4:** Clinical characteristics of, MI + DM + HTN patients infected with COVID-19.

	MI + DM + HTN + COVID-19	MI + DM + HTN	Control	*p-*value
M/F	12/15	13/17	10/12	
M/F (%)	44.4/55.6	43/57	45.5/54.5	
Age (Year)	72.42 ± 13.76 (35, 80)	57.54 ± 17.55 (31,80)	63.45 ± 20 (31,82)	^a^0.41,^b^0.83
Systolic (mmHg)	198.57 ± 8.48 (190, 210)	193.57 ± 13.15 (160,210)	118.64 ± 9.90 (105,135)	^a^0.00^a^,^b^0.00^a^
Diastolic (mmHg)	100.71 ± 4.24 (95, 105)	102.14 ± 8.28 (90,115)	81.59 ± 5.85 (70,90)	^a^0.00^a^,^b^0.00^a^
D Dimer (µg/mL)	1.54 ± 0.09 (1.30,2.90)	0.38 ± 0.41 (0.17,2.50)	0.26 ± 0.07 (0.19,0.38)	^a^0.00^a^,^b^0.63
Ferritin (ng/mL)	861.43 ± 118.58 (690, 1,003)	291.25 ± 97.77 (130,439)	247.00 ± 46.25 (187,309)	^a^0.00^a^,^b^0.19
RBS (mg/dL)	487.86 ± 59.99 (442, 582)	438.54 ± 67.59 (319,561)	159.32 ± 26.93 (123,197)	^a^0.00^a^,^b^0.00^a^
FBS (mg/dL)	187.43 ± 12.15 (170, 204)	200 ± 7 (189,209)	89.68 ± 7.23 (72,108)	^a^0.00^a^,^b^0.00^a^
HbA1c (%)	9.30 ± 1.66 (7, 13)	10.09 ± 2.10 (7,14.10)	5.05 ± 0.16 (4.80,5.40)	^a^0.00^a^,^b^0.00^a^
Blood Urea (mg/dL)	38.43 ± 20.95 (21, 79)	39.79 ± 17.60 (19,66)	37.09 ± 21.19 (24,70)	^a^1.00,^b^1.00
CRP mg/L	98.89 ± 4.35 (87, 110)	3.86 ± 1.09 (2.1,5.90)	2.52 ± 0.39 (1.80,3)	^a^0.00^a^,^b^0.21
S LDH U/L	970.29 ± 46.95 (918, 1,028)	300.18 ± 101.28 (132,540)	277.59 ± 30.62 (197, 304)	^a^0.00^a^,^b^0.47
Creatinine (mg/dL)	2.19 ± 1.15 (0.79, 4)	0.91 ± 0.42 (0.39,1.7)	0.92 ± 0.45 (0.40,1.70)	^a^0.00^a^,^b^1.00
ALP (U/L)	114.71 ± 12.48 (97, 142)	112.29 ± 12.42 (101,129)	113.18 ± 12.80 (105,139)	^a^1.00,^b^1.00
Bilirubin (mg/dL)	0.66 ± 0.12 (0.54, 0.83)	0.69 ± 0.12 (0.56,0.80)	0.75 ± 0.14 (0.50,1)	^a^0.98,^b^1.00
SGPT (U/L)	37.00 ± 12.50 (18, 58)	35.14 ± 8.48 (17,50)	36.36 ± 6.48 (20,50)	^a^1.00,^b^1.00
Hb (g/dL)	11.28 ± 1.70 (8, 13)	13.94 ± 1.48 (8.90,15.20)	14.17 ± 1.46 (11.70,16.70)	^a^0.00^a^,^b^0.84
TLC (×10^9^/L)	13.64 ± 2.00 (12, 19)	6.44 ± 2.40 (3.2,13.10)	8.68 ± 1.32 (7,11)	^a^0.00^a^,^b^0.63
RBC (×10^12^/L	4.34 ± 0.55 (4, 5.21)	4.33 ± 0.55 (3.3,5.20)	4.27 ± 0.51 (3.90,5.18)	^a^1.00,^b^1.00
Platelet (×10^9^/L)	193.12 ± 46.22 (131, 290)	216.93 ± 60.86 (122,308)	258.40 ± 52.64 (141,310)	^a^0.00^a^,^b^0.07
Lym (%)	12 ± 2.80 (9, 25)	27.43 ± 6.60 (17,40)	32.60 ± 6.23 (18,41)	^a^0.00^a^,^b^0.72
Mon (%)	0.86 ± 0.65 (0.00, 2)	2.25 ± 0.83 (1,4)	5.41 ± 1.05 (4,7)	^a^0.00^a^,^b^0.00^a^
Neu (%)	86.86 ± 3.85 (84, 88)	65.89 ± 6.75 (53,76)	57.13 ± 5.17 (51,71)	^a^0.00^a^,^b^0.26
Bas (%)	0.57 ± 0.50 (0.00, 2)	1.90 ± 1.15 (0,5)	2.22 ± 0.50 (1,4)	^a^0.00^a^,^b^0.83
Eos (%)	0.71 ± 0.46 (0.00, 1)	2.64 ± 1.26 (0,5)	2.23 ± 0.87 (1,4)	^a^0.00^a^,^b^0.83
NLR	7.46 ± 1.11	2.60 ± 0.86	1.85 ± 0.60	^a^0.00^a^,^b^0.09
NMR	80.71 ± 15.70	34.85 ± 17.07	11.04 ± 2.87	^a^0.00^a^,^b^0.00^a^

δ ANOVA, followed by Tukey’s multiple comparison test.

^a^ *p*-value was significant when less than 0.05. Data is presented as mean ± SD.

^b^ Disease with COVID-19 Vs. Control.

^c^ Disease without COVID-19 Vs. Control.

### Hematological changes

Hemoglobinopathy may seriously compromise the capacity of erythrocytes to transport oxygen under hypoxia. Therefore, low levels of oxygen were observed in different patients with COVID-19. The Hb level was observed low in patients with COVID-19 only, and solo disease conditions of DM and HTN with COVID-19 infection (11.61 ± 1.80, 11.5 ± 2.13, and (11.88 ± 1.47 g/dL). In addition, different viral infections, including COVID-19, also cause thrombocytopenia. However, thrombocytopenia was observed in the present disease group of patients with COVID-19 (161.43 ± 30, 154.6 ± 43.88, 168.3 ± 49.2/uL) compared with the control. Hemophagocytic lymphohistiocytosis disorder is widely associated with different species of viral infections, including COVID-19. Lymphopenia was noted in the disease groups of COVID-19 patients with respective disease conditions (9.44 ± 2.95, 13.06 ± 3.80, and 12.94% ± 2.50%). The level of monocytes was significantly low in the present group of patients with COVID-19 (3.14 ± 1.77, 3.21 ± 2.15, and 1.59% ± 0.50%). The levels of basophils and eosinophils were also significantly low in the COVID-19 patients in the respective disease group. Neutrophils play an essential role in clearing pathogens and debris through phagocytosis. However, its level also increases during COVID-19 infection. The elevated levels of neutrophils were observed in different patients with COVID-19 only and solo disease conditions of DM and HTN (86.67 ± 3.56, 82.31 ± 3.29, and 83.12% ± 2.26%). The NLR data showed significant increased by 10.19 ± 3.46, 6.91 ± 2.13, and 6.67 ± 1.29. In addition, the NMR was also significantly high 37.87 ± 21.63, 31.97 ± 10.74, and 59 ± 21.74 in the present disease group of COVID-19 (Table 1).

The Hb level was also found to be low in the group of COVID-19 patients with solo disease condition of IHD and MI (10.83 ± 1.85 and 11.441.68 g/dL). Platelets play an essential role in inflammatory signaling and the infectious response. However, the level of platelets was also low within the normal limit, at 173.55 ± 41.00 and 171.1 ± 50.5/ul. In addition, lymphopenia was also observed in the present solo disease conditions of IHD and MI infected with SARS-CoV-2 (13.27% ± 4.40% and 9.24% ± 2.98%). The level of monocytes was also significantly low in the COVID-19 disease group of IHD and MI (2.07% ± 0.77% and 3% ± 1.82%). The SARS-CoV-2 patients with respective disease conditions also showed low levels of basophils and eosinophils. However, the neutrophil level was also significantly high in the IHD and MI groups of COVID-19 patients, with 83.07% ± 4.04% and 87.12% ± 3.33%. The NLR was significantly high in the present population of patients with disease (7.61 ± 4.19 and 10.43 ± 3.4. In addition, the NMR was significantly high 46.50 ± 19.47 and 41.23 ± 23.8 shown in Table 2.

The Hb level was low in different patients with SARS-CoV-2 with multiple disease conditions of IHD with DM, DM with HTN, MI with HTN, and MI and DM 11.00 ± 1.34, 11.69 ± 1.35, 11.16 ± 1.40, and 11.83 ± 1.53 g/dL, respectively. In addition, low platelet levels were also noted in the present group of COVID-19 patients (160.10 ± 49.00, 177.80 ± 49.00, 161.70 ± 33.70, 175.10 ± 58.60/µL). The SARS-CoV-2 patients in the present disease group also showed lymphopenia (13.50 ± 2.37, 18.42 ± 19.48, 12.92 ± 1.96, 13.25% ± 2.45%). However, a low level of monocytes was also observed in SARS-CoV-2 patients with the respective disease conditions. In addition, basophil and eosinophil levels were also low in different COVID-19 patients in the present disease group. A significantly high level of neutrophils was also noted in COVID-19 patients with the respective multiple disease conditions (83.50 ± 2.04, 85.08 ± 2.42, and 85.25% ± 2.15% and 84.25% ± 2.65%). In addition, NLR was significantly elevated at 6.40 ± 1.27, 6.77 ± 1.43, 6.76 ± 1.09 and 6.59 ± 1.30. However, NMR was also significantly elevated at 59.50 ± 20.44, 70.96 ± 20.54, 78.08 ± 16.00 and 80.67 ± 11.77 (Table 3).

The patients with SARS-CoV-2 with multiple disease condition of MI, DM, and HTN also showed low levels of Hb. The present group of COVID-19 patients with respective diseases also showed low levels of platelets (193.12 ± 46.22). In addition, the COVID-19 patients in this group also showed lymphopenia (12.00% ± 2.80%). Moreover, the monocytes and basophils levels were also low in the present COVID-19-infected group, with respective disease 0.86% ± 0.65% and 0.57 ± 0.50. Low levels of basophils and eosinophils were also observed. However, a significantly elevated neutrophil count (86.86% ± 3.85%) was also observed. The NLR was significantly high in the present group of patients with 7.46 ± 1.11, while the NMR was also significantly increased by 80.71 ± 15.70, as shown in Table 4.

## Discussion

SARS-CoV-2 can affect various physiological systems, including the blood and immune system. The SARS-CoV-2 primarily targets the respiratory system (Rahi et al., 2021). However, the infection can have systemic effects. The impact on serum and hematological parameters can vary among individuals and is influenced by factors such as age, overall health, and the severity of the infection (Bellmann-Weiler et al., 2020; Higgins et al., 2020). This statement agrees with our results, which demonstrate that SARS-CoV-2 infection significantly modulates serum and hematological parameters in the existing health condition.

The elevated levels of liver enzymes are frequently observed by different physicians during their clinical practice. Therefore, it can be difficult to evaluate such problems in people with no symptoms. In addition, liver enzymes are also elevated during different hepatitis infections, fatty liver disease, obesity and hemochromatosis, etc (Malakouti et al., 2017). Kidney failure may also occur during chronic DM and may elevate the levels of urea and creatinine in blood (Dabla, 2010). Furthermore, high levels of urea and creatinine are also found during glomerulonephritis, use of certain drugs and consuming large amounts of protein diets, *etc.* It indicates that it may be difficult to identify the cause of elevated levels of liver enzymes, urea and creatinine. Therefore, in the present cohort study we selected precise markers that modulate during SARS-CoV-2 infection. The abnormal coagulation during COVID-19 results in the elevated level of D-dimer (Han et al., 2020). The clinical investigations to diagnose the deep vein thrombosis or pulmonary embolism suggested that the elevated level of D-dimer directly linked with the risk of irregular blood clotting (Querol-Ribelles et al., 2004; Yao et al., 2020). However, several studies depicted 3 to 4-folds increase in D-dimer levels to be associated with the better prognosis in COVID-19 patients. However, in the present study elevated D-dimer levels have also been identified in solo and multiple disease conditions of DM, HTN, IHD and MI infected with COVID-19. Therefore, the early stages of the disease’s coagulation parameters can also help to prevent and treat COVID-19 infection. The detailed investigation of serum ferritin as a marker of iron metabolism demonstrated that the changes in its concentrations is one of the systemic symptoms of the acute phase reaction (de Lucena et al., 2020). Serum ferritin has been investigated for a long time as a marker of iron metabolism (Wang et al., 2010; Henry et al., 2020b; Vargas-Vargas and Cortés-Rojo, 2020). Moreover, it has been demonstrated that the high ferritin levels result due to acute inflammatory reactions. Likewise in the current investigation, the recorded serum ferritin level was significantly increased both in solo as well as multiple disease conditions of the patients infected with COVID-19. CRP level is the key indicator associated with the degree of inflammation irrespective of the age and gender (Bilgir et al., 2015). The high CRP levels can be utilized to the early diagnosis of the severe lung infections in the patients with severe pneumonia (Warusevitane et al., 2016). In the present study, the significantly increased levels of inflammatory marker such as hs-CRP were observed in all the disease conditions of COVID-19 infection that can also be used as rapid diagnostic markers for disease severity at early stages of infection. The inflammatory responses showed the association between infectious cells, the immune system, and the inflammatory response, mimicking nonspecific responses to hypoxia, tissue injury, and necrosis. Moreover, previous studies also explored that several conditions such as cancer, heart failure, hypothyroidism, and viral diseases, including COVID-19, can increase LDH levels (Henry et al., 2020a; Wu et al., 2020). In the present cohort, the LDH level was significantly increased in patients of solo and multiple disease conditions of DM, HTN, IHD and MI infected with COVID-19.

Previous studies showed that specific laboratory parameters at the time of hospitalization were linked to mortality and the severity of the COVID-19 infection (Allouche et al., 2021; Anai et al., 2021; Al-Saadi and Abdulnabi, 2022; Gajendra, 2022). However, the present findings showed a low level of Hb in COVID-19 patients at the time of admission to the ICU and were associated with disease severity (Freeman and Morando, 2018). Therefore, a decline in Hb level upon diagnosis of COVID-19 may be a sign of acute respiratory failure. Moreover, the low level of Hb was also observed in all the patients infected with COVID-19 and hence could be assigned to acute respiratory failure in COVID-19 infection. The state of leukocytosis could be substantially associated to the death of individual which can be occurred in response to the external stimuli including infection, inflammation, drugs, malignancies, poisoning and ketoacidosis. Furthermore, it is also associated with acute leukemias, chronic leukemias and myeloproliferative disorders (Asadollahi et al., 2011). Therefore, in COVID-19 infection, a higher TLC count should receive more significant consideration, which would help practitioners to take an even more effective treatment (Zhu et al., 2021). However, in the present study, elevated levels of TLC were observed in all the patients with different disease conditions infected with SARS-CoV-2. In addition, during the onset of COVID-19 infection, viral spike proteins and complement activation products have been detected on the cell surface of erythrocytes and reduce the level of RBC. However, the RBC levels were not significantly reduced in the present investigation. In the severe stage of SARS-CoV-2 infection, the increase risk of mortality is also associated with thrombocytopenia. The first cellular response to vascular injuries and thrombosis is the elevation in the levels of platelets. Hence by releasing numerous potent cytokines and chemokines, the platelets can link the immune system to thrombotic events (Franco et al., 2015). In the present finding, thrombocytopenia has been observed in all the patients infected with COVID-19. The findings of the current investigation observed thrombocytopenia in all the studied patients infected with SARS-CoV-2. Moreover, the present findings also showed significant differences for some parameters including Hb, TLC, RBC, and platelets as compared to control, although the index was in the normal range.

Hemophagocytic lymphohistiocytosis disorder is considerable associated with the several species of viral infections (Mostaza-Fernández et al., 2014). However, differentiation in the peripheral white blood cell composition could indicate immunologic dysfunction during COVID-19 infection (Higgins et al., 2020). Likewise, neutrophilia is another sign of cytokine storm and hyper-inflammation state and hence considered as a predictor of disease severity in COVID-19 infection. (Chan et al., 2021). Therefore, neutrophilia is also considered a predictor of disease severity in COVID-19 infection. On the other side, as a poor prognostic indicator several studies also demonstrated the association of eosinophilia with COVID-19 disease severity. Severe SARS-CoV-2 patients have lower lymphocyte counts and higher leukocyte counts, identified as a prognostic indicator (Tong et al., 2021). However, elevated levels of neutrophil-to-lymphocyte ratio (NLR) and neutrophil-to-monocyte (NMR) ratio have emerged as independent hallmarks of severe COVID-19 infection (Rizo-Téllez et al., 2020; Reusch et al., 2021). In the present cohort, lymphopenia, monocytopenia, neutrophilia, eosinopenia, and basopenia have been observed in all the patients with solo and multiple disease conditions of DM, HTN, IHD and MI. In addition, high NLR and NMR were also observed in all the respective disease conditions infected with Severe SARS-CoV-2.

## Conclusion

This study provides evidence of a relationship between COVID-19 infection and modulation in serum and hematological parameters. However, severe SARS-CoV-2 infection induces changes in the serum and hematological parameters which could be used as a marker for the rapid diagnosis of SARS-CoV-2 infection in patients with solo and multiple disease conditions of DM, HTN, IHD and MI.

## Data Availability

The raw data supporting the conclusions of this article will be made available by the authors, without undue reservation.
